# Supplementary figures and images for: Multinomial modelling of TB/HIV co-infection yields a robust predictive signature and generates hypotheses about the HIV+TB+ disease state
Source: PLoS One. 2019 Jul 15;14(7):e0219322. doi: 10.1371/journal.pone.0219322 (PMC6629068; doi:10.1371/journal.pone.0219322)

six.rf.10 on validation sets: TB vs non-TB

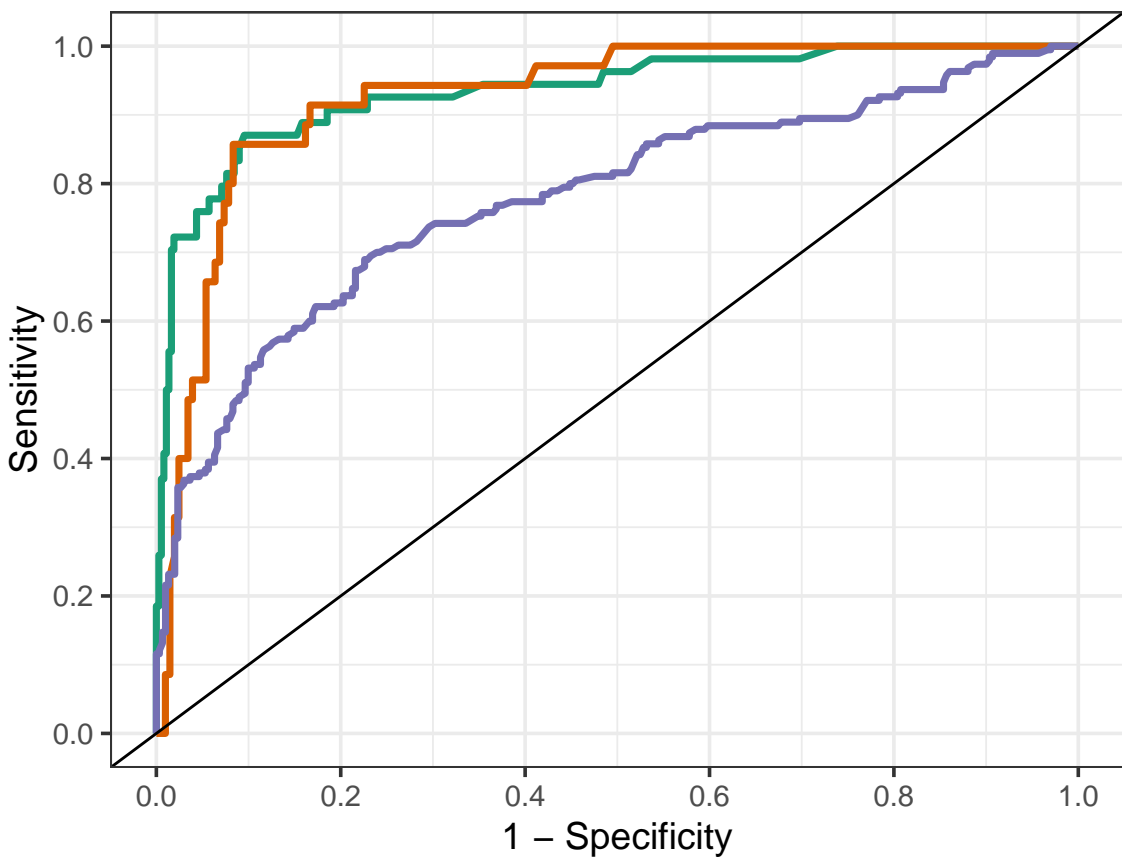

Berry: GSE19491, AUC:0.94(0.9–0.97)  
Bloom: GSE42843, AUC:0.93(0.88–0.97)  
Anderson: GSE39941, AUC:0.78(0.73–0.82)

Supplement: S1 Fig — (PDF) [file pone.0219322.s001.pdf]
