# Supplementary material for: Multinomial modelling of TB/HIV co-infection yields a robust predictive signature and generates hypotheses about the HIV+TB+ disease state
Source: PLoS One. 2019 Jul 15;14(7):e0219322. doi: 10.1371/journal.pone.0219322 (PMC6629068; doi:10.1371/journal.pone.0219322)
Supplement: S2 File — (DOCX) [file pone.0219322.s003.docx]

## Additional File 2: Key to model structure names

Models trained in the manuscript are named in the form

<classes-predicted>.<algorithm>.<number-of-probes>

where classes-predicted is one of the below options

| **Classes Predicted** | **Description** |
| --- | --- |
| six | Six-class multinomial model, predicts the following classes  [TB.HIV+, TB.HIV-, LTB.HIV+, LTB.HIV-, OD.HIV+, OD.HIV-] |
| four | Four-class multinomial model, predicts the following classes  [TB.HIV+, TB.HIV-, LTB.HIV+, LTB.HIV |
| twopos | Two-class binary model, predicts TB or LTB, trained on HIV+ samples only |
| twoneg | Two-class binary model, predicts TB or LTB, trained on HIV- samples only |

and algorithm is from one of the below options

| **Algorithm** | **Description** |
| --- | --- |
| glmnet | Logistic regression with elastic-net regularization (L1 and L2) |
| knn | K-nearest neighbours |
| nnet | Neural network |
| rf | Random forest |
| svmRadial | Support vector machine with radial basis function kernel |
| dnn | Stacked autoencoder deep neural network |
| xgbTree | Extreme gradient boosting |

Thus, the model named **six.rf.25** indicates a six-class multinomial random forest model based on 25 microarray probes.

For comparison, two external models have been included, named as follows

| **External Model Name** | **Description** |
| --- | --- |
| threeGene | Three-gene TB diagnostic signature, published by Sweeney et al (2016), consisting of the genes *GBP5*, *KLF2* and *DUSP3*. |
| ACS | Signature of risk TB progression derived from South African adolescents with latent TB. Based of splice-junctions expression from 16 genes. |
